# Temporal genetic diversity of *Schistosoma japonicum* in two endemic sites in China revealed by microsatellite markers

**DOI:** 10.1186/s13071-016-1326-7

**Published:** 2016-01-22

**Authors:** Mingbo Yin, Hongyan Li, David Blair, Bin Xu, Zheng Feng, Wei Hu

**Affiliations:** School of Life Sciences, Fudan University, 2005 Songhu Road, Shanghai, 200438 China; College of Marine and Environmental Sciences, James Cook University, Townsville, Qld 4811 Australia; National Institute of Parasitic Diseases, Chinese Center for Disease Control and Prevention, 207 Ruijin Er Road, Shanghai, 200025 China

**Keywords:** *Schistosoma japonicum*, Genetic diversity, Population structure, Temporal variation, Microsatellite

## Abstract

**Background:**

Schistosomiasis is one of the neglected tropical diseases. The causative agent of schistosomiasis in China, *Schistosoma japonicum*, has long been a major public health problem. An understanding of fundamental evolutionary and genetic processes in this species has major implications for its control and elimination. Intensive control efforts have greatly reduced the incidence of schistosomiasis in China, but little is known about the genetic consequences of these efforts.

**Methods:**

To investigate this, we sampled twice (years 2003 and 2011) from two endemic regions where populations of *S. japonicum* had persisted despite control efforts and genotyped these samples using ten microsatellite markers. Our main hypothesis was that parasite genetic diversity would be greatly reduced across this time period.

**Conclusions:**

There was no apparent reduction in allelic diversity, and a non-significant reduction in clonal diversity in these parasite populations between 2003 and 2011. We did, however, detect temporal genetic differentiation among the samples. Such a significant temporal genetic variation of *S. japonicum* populations has not been reported before.

## Introduction

Schistosomiasis is a serious parasitic disease, infecting over 200 million people and threatening the health of about 779 million people around the world [1]. Schistosomiasis due to infection with *Schistosoma japonicum* has long been a major public health problem in China [2]. In the last decade, the disease has been intensively controlled in most endemic regions in China [3]. Specifically, in 2004, a national program for schistosomiasis control was launched, aiming to reduce the transmission of *S. japonicum* from cattle and humans to snails. Effective interventions were implemented from 2005 to 2007, including keeping cattle away from snail-infested grasslands, providing farmers with mechanised farming tools and improving public environmental sanitation by supplying tap water and building lavatories. Annual synchronous rounds of chemotherapy are also routinely used [4]. It has been proposed that evolutionary theory should have an important role in the design, application and interpretation of such programs [5]. Control interventions are expected to reduce the genetic diversity within the parasite population. Associated with this, intensive selection is likely, driving rapid evolutionary changes in natural parasite populations [5]. For example, under strong drug selective pressure, resistance could emerge and become fixed, since the drug-resistant phenotypes and alleles have an advantage over the wild type in these adverse circumstances [5]. Although we cannot test for such selection here, we can look for evidence of reduced genetic diversity in parasite populations following intervention.

Genetic diversity is of great importance for any organism to adapt in a changing environment and, in the case of parasites, to respond to the pressure of intervention programs [6]. Different types of molecular markers, for instance, mitochondrial DNAs [7] and microsatellites [8], have been applied to evaluate genetic variation in *S. japonicum* populations among different geographical regions [9], as well as the genetic differentiation among host species [10] or host individuals [11]. Some recent studies have reported spatial genetic variation [10, 12, 13] among natural *S. japonicum* populations. There have also been reports on spatio-temporal modeling for the prevalence of *S. japonicum* infection [14, 15]. However, to date, no study has investigated temporal changes in genetic structure of *S. japonicum* populations.

In this study, we sampled again in 2011 from seven endemic regions where *S. japonicum* populations were present in 2003. In 2011, the parasite was only detected in two of the seven locations, suggesting a high degree of intervention success at several locations. For the two locations at which parasites had persisted, we hypothesised that a considerable reduction in genetic diversity (allelic richness and clonal diversity) would have occurred between these time points as a consequence of the interventions. In addition, we applied a range of population-genetic analyses under the hypothesis that there should be no temporal changes between the time points.

## Methods

### Ethics statement

All procedures involving animals were carried out according to the guidelines of the Association for Assessment and Accreditation of Laboratory Animal Care International. Our protocol followed institutional ethical guidelines that were approved by the ethics committee at the National Institute of Parasitic Diseases, Chinese Center for Disease Control and Prevention (NIPD, China CDC; Permit No: IPD2008-4).

### Sample collections

In 2011, we re-sampled seven endemic sites in China that had been investigated in 2003 for our previous project [16]. In the interim, the National Program for Schistosomiasis Control had reduced the transmission of *S. japonicum* [17] to such an extent that infected snails were detected in only two out of seven locations: Yueyang city in Hunan Province and Shashi city in Hubei Province (Table 1). These two sites are both close to the Yangtze River and about 129 km apart.

**Table 1:** Sampling information and genetic diversity of *Schistosoma japonicum* subpopulations

Locality (Abbreviation)	Latitude/Longitude	Year	Gender	Abbreviation of subpopulation	Number of individuals		Clonal diversity (R)		Number of unique MLGs		Number of MLGs (after removing near-identical MLGs)		*H*_*E*_	*H*_*O*_	*A*_*r*_
Hunan Province, Yueyang City (YY)		2003	F	YY03F	51	28	0.98	1	50	28	33	19	0.90	0.78	10.93
	29.33 N		M	YY03M		23		0.95		22		14			
	113.06 E	2011	F	YY11F	44	23	0.7	1	31	23	10	7	0.86	0.81	10.40
			M	YY11M		21		0.35		8		3			
Hubei Province, Shashi City (SH)		2003	F	SH03F	23	9	0.91	1	21	9	14	6	0.84	0.83	8.36
	30.32 N		M	SH03M		14		0.85		12		8			
	112.35 E	2011	F	SH11F	44	22	0.81	0.67	36	15	25	12	0.90	0.85	10.80
			M	SH11M		22		0.95		21		13			

MLG, number of multilocus genotypes; *H*_*E*_, expected heterozygosity; *H*_*O*_, observed heterozygosity; *A*_*r*_, allelic richness

Ten laboratory-raised Kunming mice were each infected with about 1,000 *S. japonicum* cercariae isolated from infected snails (*Oncomelania hupensis*) collected from two locations (i.e. Yueyang and Shashi) in 2011. For each location, we screened 5,000 snails in 2011 and detected three infected snails from Shashi and 25 infected snails from Yueyang. After 45 days, adult worms were obtained by perfusing the hepatic portal system and mesenteric veins of each infected mouse. All worms were washed at least three times with normal saline (0.9 % NaCl) to remove the host tissues before being stored in 95 % ethanol at 4 °C. The gender of each worm was noted.

### DNA extraction, PCR amplification and microsatellite genotyping

In this study, we randomly selected 44 worms per location from the year 2011 samples for the genetic analyses. Genomic DNA was extracted individually from adult schistosomes using a DNeasy Blood & Tissue Kit (QIAGEN, Germany), following the manufacturer’s instructions, and then stored at -20 °C until use.

The DNA of each individual *S. japonicum* was genotyped at ten microsatellite loci (i.e. Sjp1, 4, 5, 6, 8, 9, 10, 14, 15 and 17) from our previous work [16]. The PCRs were performed in a total volume of 12 μl following the protocol in [16]. Then the PCR products were diluted to appropriate concentrations and analysed on an ABI 3730 capillary automated sequencer using a LIZ 500-labeled size standard. Allele sizes were read using GeneMapper software (version 4.0) and checked carefully.

To compare the genetic variation of *S. japonicum* populations between 2003 and 2011, we included microsatellite data from 51 worms from Yueyang and 23 worms from Shashi, which were collected in 2003 and published previously [16]. This yielded four population categories for analysis (two per time point and per location). For some analyses, these categories were further subdivided by sex (termed subpopulations here). We excluded all individuals for which data were missing.

### Microsatellite analyses

Clonal diversity (R) was calculated manually as *R* = (G-1)/(N-1) [18], where G is the number of genotypes and N represents sample size. Clonal diversity can vary from 0 (all individuals belong to one clone) to 1 (each individual is unique). All 162 individuals were included in this analysis. Principal coordinates analyses (PCoA) were then performed to display the relatedness of all unique multi-locus genotypes (MLGs) in GenAlEx 6.5 [19]. Somatic mutations occurring during the proliferation of cercariae from a single miracidium may produce multiple near-identical MLGs [16]. Inclusion of clusters of near-identical siblings may bias results. We therefore removed near-identical MLGs in the subsequent analyses, retaining only a single representative of each cluster [20]. PCoAs were then done to show the relatedness of the 82 retained MLGs. All subsequent analyses were based on these 82 MLGs. Genetic diversity indices (allelic richness *A*_*r*_; sample size was corrected) were estimated using FSTAT V2.9.3.2 [21]. As the sample size was low, males and females were not treated separately in the calculations of allelic richness.

Genetic differentiation between pairs of samples was calculated as Wright’s *F*_*ST*_ in Arlequin 3.11 [22], and the significance of the *F*_*ST*_ value was tested using 10^4^ permutations. Hierarchical analysis of molecular variance (AMOVA) was applied to quantify genetic variances into spatial, temporal and within-sample components in Arlequin 3.11.

## Results

**Fig. 1:**
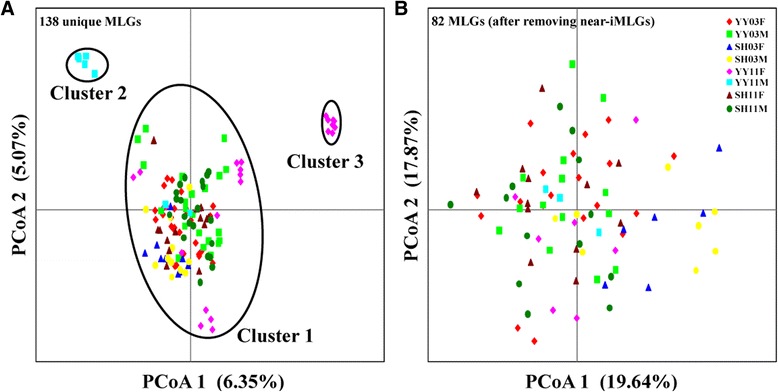
Principal coordinates analyses (the first two factors), based on co-dominant alleles at ten microsatellite loci, displaying genetic similarities among individuals of *Schistosoma japonicum* sampled from two locations and both genders at two time points (2003 and 2011) with all 138 unique MLGs (**a**), and retaining only a single representative of each near-identical MLG cluster (82 MLGs) (**b**). The abbreviated name of each subpopulation is described in Table 1

In total, 138 unique MLGs were detected among 162 *S. japonicum* individuals based on ten microsatellite loci. No shared MLG was found among the eight subpopulations (location, year and gender). The PCoA revealed that the 138 unique MLGs fell into a main cluster (Cluster 1) with small outlying clusters (Fig. 1a). Most (6 of 8) MLGs in the male subpopulation from Yueyang in 2011 (YY11M) were assigned to a small cluster (Cluster 2), whereas the other two were in Cluster 1 (Fig. 1a). Additionally, some (8 of 23) unique MLGs in the female subpopulation from Yueyang in 2011 (YY11F) formed another small cluster (Cluster 3) while the remainder fell into Cluster 1 (Fig. 1a). Overall, 56 individuals exhibited near-identical MLGs (Table 1), indicating that they might be siblings. When only a single representative of each near-identical MLG group was retained, no visually obvious temporal or spatial structuring was apparent among the remaining 82 MLGs (Fig. 1b).

Importantly, allelic richness among the 82 retained MLGs and corrected for sample size did not change dramatically at either site between 2003 and 2011 (Table 1). Indeed, allelic richness at Shashi was greater in 2011 than in 2003. The clonal diversity (R) of subpopulations ranged from 0.35 to 1.00 (average *R* = 0.85). The value of 1.00 (detected in three subpopulations; Table 1) indicated that all the individuals possess different MLGs. The clonal diversity of the 2011 male subpopulation in Yueyang was particularly low (0.35), compared with 0.95 in 2003. By 2011, the overall clonal diversity had decreased (average *R* = 0.76), but not significantly (*P* = 0.06).

Genetic differentiation was significant for all tested pairs of four *S. japonicum* samples and the *F*_ST_ values ranged from 0.009 to 0.046 (averaged over all loci, Table 2). AMOVA analyses indicated that within-population variance explained most of the observed genetic variation (97.45 %). The remaining genetic variation was due to additional variance across space (0.02 %) and across time within space (2.53 %; Table 3). The temporal component was significant but spatial was not (Table 3).

**Table 2:** Pairwise genetic differentiation (*F*_*ST*_) among four populations of *Schistosoma japonicum* based on 82 unique multi-locus genotypes (MLGs). The abbreviated name of each subpopulation is described in Table 1. All values are significant below the level of 0.05

	YY03	YY11	SH03	SH11
YY03				
YY11	0.020			
SH03	0.034	0.046		
SH11	0.009	0.020	0.041

**Table 3:** Analysis of molecular variance (AMOVA) among sampling sites and within time points of *S. japonicum*. Based on 82 unique multi-locus genotypes (MLGs)

Source of variation	d.f.	Sum of squares	Percentage of variation (%)	*P*
Across space	1	7.49	0.02	0.34
Across time (within space)	6	40.47	2.53	<0.001
Within samples	156	704.34	97.45	<0.001

## Discussion

Using microsatellite markers, we noted a non-significant decrease in clonal diversity between the two time periods, but no obvious change in allelic richness. Measures of gene flow and population structure indicated substantial temporal genetic change (between 2003 and 2011) according to population differentiation tests (*F*_ST_) and the AMOVA. Spatial structure (between Yueyang and Shashi) was also indicated according to *F*_ST_ values, but not according to the AMOVA results.

Clonal diversities were in agreement with previous studies using microsatellites [8, 20]. A high clonal diversity (average *R* = 0.94) was detected in *S. japonicum* populations from the year 2003. However, by 2011, this had decreased but not significantly. The clonal diversity in subpopulation YY11M was very low (0.35), perhaps indicating that the sample contained many sibling cercariae from a single male miracidium. Such a temporal change in clonal diversity of *S. japonicum* populations has not been observed previously.

The maintenance of high allelic richness across time was not expected. Infected snails were more difficult to find in 2011 than in 2003 (and indeed were not found at all in five of seven locations in 2011). This suggests a reduction in overall population size of *S. japonicum* in the region, but apparently not to an extent sufficient to impact markedly on allelic richness. Similarly, the extent of temporal population-genetic structure was unexpected. Such temporal changes are not previously known for *S. japonicum*, although structure at small spatial scales has been reported [8, 11, 16]. Little is known about the metapopulation dynamics of *S. japonicum* over the vast region of the mid-Yangtze basin, from which our samples came. Local extinction followed by repopulation from elsewhere in the region might partly explain the extent of genetic differentiation observed. Given the known fluxes of water in the region [23], especially in connection with annual floods, there may be a large regional population of *S. japonicum* subject to substantial dispersal and gene flow.

At our study locations, intensive control measures (annual chemotherapy) had been implemented during the investigation period (spanning 9 years), which might have acted as a strong selective pressure and been responsible for the temporal changes observed. There were also marked ecological changes during this period, in particular the completion of the Three Gorges Dam (TGD), which significantly reduced the amount of water reaching our sampling locations (Dongting and Poyang lakes) and has moderated the annual floods. Given the lack of baseline data from prior to the construction of the TGD, and the lack of understanding of metapopulation dynamics in *S. japonicum*, few strong conclusions can be drawn.

There are several limitations of our study. One is the use of different mammal hosts between 2003 (rabbits) and 2011 (mice). LoVerde *et al.* [24] noted that use of rodents (as opposed to primates) as experimental hosts led to increased reduction in genetic diversity over several generations in *S. mansoni*. However, we used mammal hosts only for a single passage (field-collected snails were used to infect laboratory hosts from which adult worms were harvested) and not serial passages such as used by LoVerde *et al.* [24]. In effect, our worms are analogous to those subjected to electrophoresis from the M_0_ generation of laboratory hosts, in which very little deviation from the parental population was observed [24]. Another limitation relates to the small number of infected snails (three) found in Shashi in 2011. Despite this, the clonal and allelic diversities within the parasite population there were high. The inference is that at least one of the infected snails had been invaded by multiple unrelated miracidia. A further point to consider is that adult *S. japonicum* can be long-lived in the mammal host, 47 years being reported in one human case [25]. Long-lived individuals can carry substantial genetic diversity through a bottleneck event. The interventions have taken place over a relative handful of years, perhaps insufficient time for any genetic bottleneck to have caused genetic erosion. Similarly, the effects of regional ecological change, such as caused by the TGD, may take more time to become apparent in the genetic structure of schistosome populations.

In conclusion, using ten microsatellite loci, we rejected the hypothesis that genetic diversity in two *S. japonicum* populations would be reduced following control programs between the years of 2003 and 2011. We did, however, find significant genetic differentiation across time in these populations. Overall, our findings provide a deeper understanding of molecular epidemiology and population genetics of *S. japonicum,* which may be of value for effective control and elimination of schistosomiasis.
